# A conserved haplotype controls parallel adaptation in geographically distant salmonid populations

**DOI:** 10.1111/j.1365-294X.2011.05305.x

**Published:** 2012-01

**Authors:** MICHAEL R MILLER, JOSEPH P BRUNELLI, PAUL A WHEELER, SIXIN LIU, CAIRD E REXROAD, YNIV PALTI, CHRIS Q DOE, GARY H THORGAARD

**Affiliations:** * Institute of Molecular Biology and Howard Hughes Medical Institute, University of OregonEugene, OR 97403, USA; † School of Biological Sciences and Center for Reproductive Biology, Washington State UniversityPullman, WA 99164, USA; ‡ National Center for Cool and Cold Water Aquaculture, Agricultural Research Service-USDAKearneysville, WV 25430, USA

**Keywords:** adaptation, conservation, genomics, salmon, salmonids, trout

## Abstract

Salmonid fishes exhibit extensive local adaptations owing to abundant environmental variation and precise natal homing. This extensive local adaptation makes conservation and restoration of salmonids a challenge. For example, defining unambiguous units of conservation is difficult, and restoration attempts often fail owing to inadequate adaptive matching of translocated populations. A better understanding of the genetic architecture of local adaptation in salmonids could provide valuable information to assist in conserving and restoring natural populations of these important species. Here, we use a combination of laboratory crosses and next-generation sequencing to investigate the genetic architecture of the parallel adaptation of rapid development rate in two geographically and genetically distant populations of rainbow trout (*Oncorhynchus mykiss*). Strikingly, we find that not only is a parallel genetic mechanism used but that a conserved haplotype is responsible for this intriguing adaptation. The repeated use of adaptive genetic variation across distant geographical areas could be a general theme in salmonids and have important implications for conservation and restoration.

## Introduction

Variation in environmental conditions throughout a species range causes different individuals of the same species to experience distinct forces of natural selection. These forces can cause local populations to evolve traits that provide an advantage in the local environment regardless of their consequences in other environments. This process, referred to as local adaptation, leads to resident genotypes having a higher fitness in their local environment relative to genotypes originating in other environments (Kawecki & Ebert 2004). Biologists have long been fascinated by the possibility of identifying the genes and molecular pathways that underlie local adaptation, and modern genomic technologies are making this more possible than ever before (Stapley *et al.* 2010). Furthermore, understanding the genetic architecture of local adaptation has important implications for defining conservation units, determining management priorities and designing restoration programmes for threatened or endangered species (Waples 1991; Fraser & Bernatchez 2001; Taylor *et al.* 2011).

The consideration and characterization of local adaptation in salmonid fishes (which include salmon, trout, char, freshwater whitefish and graylings) is an important and long-standing area of research (Ricker 1972; Taylor 1991). Many salmonid species exist over broad and highly variable geographic areas. The combination of abundant environmental variation and their renowned ability for precise natal homing creates a situation ripe for local adaptation (Quinn 2005). Numerous examples of phenotypic variation and local adaptation have been reported within salmonid species (Halupka *et al.* 2003; Fraser *et al.* 2011), and failed restoration attempts are thought to be caused by inadequate adaptive matching of translocated populations to their new environment (Allendorf & Waples 1996).

The genus *Oncorhynchus* (meaning ‘hook-nose’) is an iconic species group that includes Pacific salmon and trout. *Oncorhynchus* species have great cultural, economic, symbolic and recreational importance in the Pacific Northwest and other regions in their native range (National Research Council 1996). Within *Oncorhynchus*, the *O. mykiss* species encompasses both resident-freshwater and anadromous (ocean-dwelling but freshwater-spawning) forms that are referred to as rainbow trout and steelhead, respectively. *O. mykiss* are native to the Pacific coast of North America from Baja California to the Alaska Peninsula and the Kamchatka Peninsula of Russia and natural populations contain diverse phenotypic adaptations (Hershberger 1992; Taylor *et al.* 2011). As with other *Oncorhynchus* species, *O. mykiss* are threatened, endangered or extinct throughout much of the native range, and restoration is considered a challenging but crucial priority (Busby *et al.* 1996; National Research Council 1996; Gustafson *et al.* 2007). Besides the importance of natural populations, ease of culture and experimental tractability have made *O. mykiss* an important species for biomedical research and aquaculture, and more is known about the biology and physiology of *O. mykiss* than about any other fish species (Thorgaard *et al.* 2002). This unique combination of attributes makes *O. mykiss* a powerful and tractable system for investigating the genetic architecture of local adaptation in salmonids.

Several clonal lines of *O. mykiss* have been established using the chromosome manipulation methods of andro- and gynogenesis (Young *et al.* 1996). The source populations for these clonal lines originate from diverse geographical regions and exhibit abundant phenotypic variation. Source population phenotypes are accurately captured by the clonal lines (Robison & Thorgaard 2004), and the lack of genetic variation within each line results in phenotypic stability across generations (Young *et al.* 1996). These lines provide valuable experimental uniformity that facilitates the identification of phenotypic variation (Ristow *et al.* 1995; Robison *et al.* 1999). Furthermore, the lines are readily amenable to various crossing schemes and chromosomal manipulations, which makes them a powerful system for the genetic dissection of identified phenotypic variation (Robison *et al.* 2001; Sundin *et al.* 2005; Nichols *et al.* 2007, 2008).

Rate of development is a fascinating trait that varies across natural populations of *O. mykiss* and other salmonid species (Robison & Thorgaard 2004). Juvenile salmonids suffer high rates of mortality after emerging from their gravel nests (Elliott 1989) and experience intense selection to optimize emergence timing for increased food availability, reduced predation rates and ideal migration conditions (Einum & Fleming 2000; Sundström *et al.* 2005). Development rate also varies across the *O. mykiss* clonal lines in a manner reflecting their source populations (Robison & Thorgaard 2004), with two lines, referred to as Clearwater (Cl) and Swanson (Sw), having an accelerated development rate relative to the others (Robison *et al.* 1999; Sundin *et al.* 2005). Interestingly, these two lines originate from very distant geographical locations and belong to distinct subspecies of *O. mykiss*. The Cl line originated near the extreme east of the *O. mykiss* native range (North Fork Clearwater River, North Central Idaho) and belongs to the inland subspecies, whereas Sw originated near the extreme north (Swanson River, Southcentral Alaska) and belongs to the coastal subspecies (Allendorf & Utter 1979; Behnke & Tomelleri 2002; Brunelli *et al.* 2010). The parallel adaptation of an increased development rate may have evolved in response to the cold incubation temperatures at both locations.

The genetic basis of development rate variation has been examined in crosses between each of the rapid-developing lines and one slower-developing line that is referred to as OSU. Interestingly, both OSU × Sw and OSU × Cl crossing schemes revealed a major quantitative trait locus (QTL) that controlled up to 30% of the observed development rate variation and localized to the same genomic region (Robison *et al.* 2001; Sundin *et al.* 2005; Nichols *et al.* 2007). Although the most parsimonious explanation for these results is that the OSU allele has a constant effect of slower development in both crosses (Nichols *et al.* 2007), the uniqueness of rapid development among the lines evokes the intriguing possibility that the indistinguishable QTL mapping occurred because the Cl and Sw populations have evolved an increased rate of development through parallel genetic mechanisms. [Correction after online publication 28 October 2011: in the preceding paragraph QTL was corrected to ‘quantitative trait locus’].

Here, we further examine the genetic architecture of development rate variation with an emphasis on investigating the potential of a parallel genetic basis underlying the parallel adaptation of rapid development in Cl and Sw. To this end, we use another slower-developing line that is referred to as Whale Rock (WR) and next-generation sequencing of restriction-site associated DNA (RAD) tags (Miller *et al.* 2007; Baird *et al.* 2008; Hohenlohe *et al.* 2010a) to identify and genotype thousands of single nucleotide polymorphisms (SNPs) in the clonal lines and mapping progeny. Strikingly, we find that not only is a parallel genetic mechanism used for rapid development in Cl and Sw but that a conserved haplotype is responsible for this intriguing parallel adaptation. In addition to providing insight into the genetic mechanisms that underlie parallel adaptation, our finding of the repeated use of adaptive genetic variation across distant geographical areas could be a general theme in salmonids and have important implications for the management and/or restoration of threatened or endangered populations.

## Materials and methods

### Fish culture and crosses

The clonal lines used in this study were originally produced from outbred populations through rounds of gyno- and/or androgenesis, are completely homozygous and are maintained at the Washington State University Trout Hatchery (Young *et al.* 1996). The Sw and Cl lines are phenotypically male and genetically YY. The WR line originated from Whale Rock Reservoir on the Central Coast of California and is phenotypically and genetically female (XX). Eggs from one WR female were fertilized by sperm from one Sw male to produce WR × Sw F1 hybrids. The hybrids are phenotypically and genetically male (XY).

To produce the doubled haploid mapping progeny, outbred eggs were gamma-irradiated to destroy nuclear DNA and fertilized with sperm from one F1 hybrid male, and the first embryonic cleavage was blocked by heat shock to restore diploidy. The outbred eggs were obtained from Troutlodge Inc. (Sumner, WA) in February 2010. Hatching time has a good concordance with other measures of ontogenesis such as enzyme expression and morphological landmarks (Ferguson *et al.* 1985) and was used as a proxy for development rate. Hatching time was measured by transferring embryos into individual wells of an 80-well box within a stack incubator, examining the embryos every 8 h and recording the time of newly hatched embryos (Robison *et al.* 1999). Hatched embryos were stored at −80 °C prior to DNA extraction.

### Molecular biology

DNA was extracted from frozen embryos or clonal line fin clips using the Qiagen DNeasy Blood and Tissue Kit according to the manufacturer’s protocol, and RNase A treatment was included. RAD libraries were constructed in a manner similar to the previously published protocols (Baird *et al.* 2008; Hohenlohe *et al.* 2010a). Our exact protocol is included in the Supporting information (Protocol S1). The Y-specific markers were genotyped as previously described (Brunelli *et al.* 2008).

The PCR amplifications and fragment size analyses for microsatellite genotyping were performed as previously described (Rexroad *et al.* 2008; Palti *et al.* 2011). For each chromosome from the genetic map of the study of Palti *et al.* (2011), 4–6 microsatellites that were mapped at high confidence and spaced at large intervals were used to screen a panel composed of the Sw and WR lines and four doubled haploid progeny. Following the screening, 2–3 polymorphic markers were used to genotype an additional 48 progeny.

### SNP discovery and genotyping

The SNP discovery and genotyping was performed using custom Perl scripts (available from the corresponding author upon request) and the alignment program Novoalign. Eighty-bp-long raw sequence reads were trimmed from the 3′ end to 71 bp, and quality scores were used to remove reads for which the probability of a sequencing error was greater than 20% or reads that contained one or more ambiguous base calls. The first 11 bp of each read were used to identify and separate the reads belonging to each sample. Only reads with an exact match to a 5-bp barcode followed by the 6-bp partial SbfI site were sorted out, and their first 11 bp were removed. We refer to these as ‘filtered’ reads.

SNPs were discovered using filtered reads from the Cl, Sw and WR clonal lines. For each line, the reads were collapsed into a FASTA-formatted file in which each unique sequence is represented once and each header line contains the clonal line name, a unique identification number and the number of read occurrences of that sequence. The three FASTA files were concatenated into a single file. We performed an alignment of the all sequences in this file using Novoalign to generate an alignment index of the file and to align the file against its own index. Novoalign was run in exhaustive mode with a maximum alignment penalty score of 250, and a maximum of 20 alignments reported. The resulting alignment output represented a map of the pairwise alignment scores for sequences within (internal alignments) and between (external alignments) the clonal lines. We used this map to group sequences into distinct loci with the following criteria: perfect internal alignments were ignored; sequences that had only one occurrence were ignored unless they had a perfect external alignment with a sequence that had more than one occurrence; alignments with a score over 90 were ignored; each locus had to contain one sequence from each line and three sequences total; and the maximum alignment score between any sequences in a locus was 30.

We genotyped the doubled haploid mapping progeny at the loci that were polymorphic between Sw and WR. A FASTA file was created that contained both Sw and WR allele sequences for all these loci. Novoalign was used to index this file and align filtered reads from each doubled haploid individual against the index. For each individual, the number of reads with a perfect match to each allele at each locus was counted. Loci in which only one parental allele was present were called the corresponding parental genotype, whereas loci for which both alleles were present were called heterozygous.

### Genetic map construction and QTL analysis

The initial genetic mapping data set consisted of 123 individuals and 4888 markers. These data were filtered prior to map construction. The doubled haploid progeny should be completely homozygous, but we identified seven individuals with high levels of heterozygosity and removed them from the data set because they most likely contained large amounts of residual maternal nuclear DNA. Likewise, we identified 81 markers with heterozygous genotype calls in five or more individuals and removed them because they most likely represented either false-positive SNPs from paralogous sequences or loci with tetrasomic inheritance that would be difficult to map. The remaining heterozygous genotype calls could be due to residual maternal nuclear DNA, barcode jumping or sequencing error, but distinguishing between these possibilities is difficult. Therefore, they were converted to missing genotypes for the purpose of map construction. We also removed four markers with extreme segregation distortion and 213 markers that were missing genotypes in greater than 25% of the individuals. The filtered data set consisted of 116 individuals and 4590 markers.

We used R/qtl (Broman *et al.* 2003) to construct a genetic map from the filtered data set. The backcross model was used to code for the doubled haploid genotypes. We initially formed linkage groups with a maximum recombination frequency of 0 and minimum logarithm of odds (LOD) score of 12 and ordered the markers using the orderMarkers function. We chose one marker with the least missing genotypes from each position and reconstructed the map with this subset of markers. We formed linkage groups with a maximum recombination frequency of 0.25 and minimum LOD score of 4. After initial ordering with orderMarkers, we used a combination of the ripple and switch.order functions to check every possible marker order in a sliding window and change the ordering when necessary. We also inspected plots of the recombination frequency and LOD scores among markers and manually changed the order when the sliding window was too small to correct ordering errors. Once the final map order had been determined, the remaining markers were added at the position of whichever marker they shared their initial ordering position with. The resulting map contained 4590 loci, consisted of 29 major linkage groups with 59–344 loci each and four small groups with 6–7 loci each, and the total size was 1837.3 cM. The small groups were discarded for the remainder of the analysis. The final map was drawn with Mapchart (Voorrips 2002).

The development rate QTL analysis was also performed using R/qtl. We used the fill.geno function to impute and replace missing genotypes for the subset of markers used in the final genetic map ordering. We next used the scanone function to scan these markers with the single QTL model for an influence on development rate. LOD scores of >3.00 were considered significant because they exceeded the 95% upper tail of the distributions generated by 1000 permutations of the data. One genomic region on linkage group 3 exceeded this threshold of significance.

## Results

### Rapid and accurate SNP discovery using next-generation sequencing

**Fig. 1:**
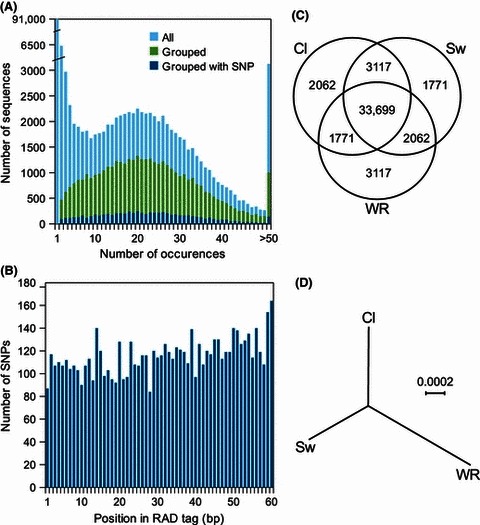
SNP discovery using next-generation sequencing. (A) Histogram showing the relationship between the number of unique restriction-site associated DNA (RAD) tag sequences and the number of occurrences for each sequence from the Sw line. Different coloured bars represent the distribution of different sets of sequences from the filtered reads. (B) Histogram showing the number of SNPs at each RAD tag sequence position for the 6950 polymorphic loci. (C) Venn diagram showing the number of unique and shared sequences among the clonal lines for the 40649 loci. (D) Unrooted tree showing the SNP frequency between lines.

To discover SNPs between the Cl, Sw and WR clonal lines, we isolated and sequenced SbfI RAD tags from each line. We generated 1.6–2.3 million filtered reads per line, which represented 144–188 thousand unique sequences (Table 1). For each line, we observed an interesting distribution in the number of times each sequence occurred among the filtered reads. The distribution had two peaks, one at a single occurrence per sequence and the other at around 20 occurrences per sequence (Fig. 1A, light blue). This pattern suggests that most of the sequences observed at a low frequency contain errors and that most error-free sequences are represented at a higher frequency. We conclude that many sequencing errors are present in the filtered reads and that the number of occurrences per sequence alone does not clearly distinguish between accurate and erroneous sequences.

**Table 1:** Restriction-site associated DNA sequencing results from the Cl, Sw and WR clonal lines

Clonal line	Filtered reads	Unique sequences	Grouped sequences	Grouped sequences with single nucleotide polymorphism
Clearwater (Cl)	2251860	187381	40649	6950
Swanson (Sw)	2123144	173158	40649	6950
Whale Rock (WR)	1664580	144506	40649	6950

To distinguish between accurate and erroneous sequences, we performed alignments between all the sequences represented in the three lines and used simple criteria (see Materials and methods) to group them into distinct loci. This grouping resulted in 40649 distinct loci, of which 6950 contained one biallelic SNP (Table 1, Appendix S1, Supporting information). Strikingly, this alignment-based grouping completely removed the peak of low-occurrence sequences while retaining most sequences represented at a higher frequency (Fig. 1A, green). The subset of higher occurrence sequences that were removed corresponds well with the large percentage of salmonid genomes that is composed of repetitive elements (de Boer *et al.* 2007; Genet *et al.* 2011). Furthermore, the distribution of SNPs was relatively flat throughout the RAD tag sequence length despite increased error rates near the end (Fig. 1B). We conclude that the vast majority of grouped sequences are nonrepetitive and error-free and that these SNPs are unlikely to represent sequencing errors.

We next examined the distribution of SNPs among the lines. Each locus contained one sequence per line, and a subset of loci contained one biallelic SNP. For each polymorphic locus, one line contained a unique sequence that differed by one SNP from a sequence shared by the remaining two. Each line had a large number of unique sequences with 2062, 1771 and 3117 from Cl, Sw and WR, respectively (Fig. 1C). This corresponds to a SNP frequency of 1 in 633 bp between Cl and Sw, 1 in 469 bp between Cl and WR and 1 in 498 bp between Sw and WR (Fig. 1D). We conclude that genetic variation is abundant and relatively evenly distributed among the lines.

This genome-wide pattern of genetic diversity differs from a previously examined Y-linked locus at which coastal subspecies (such as Sw and WR) are very similar to each other but deeply divergent from inland subspecies (such as Cl) (Brunelli *et al.* 2010). Although interesting, a single haploid genotype does not reflect the variation present in these natural populations, and future work examining genome-wide variation in natural inland and coastal populations could provide further insight.

### Next-generation sequencing facilitates rapid SNP genotyping

**Fig. 2:**
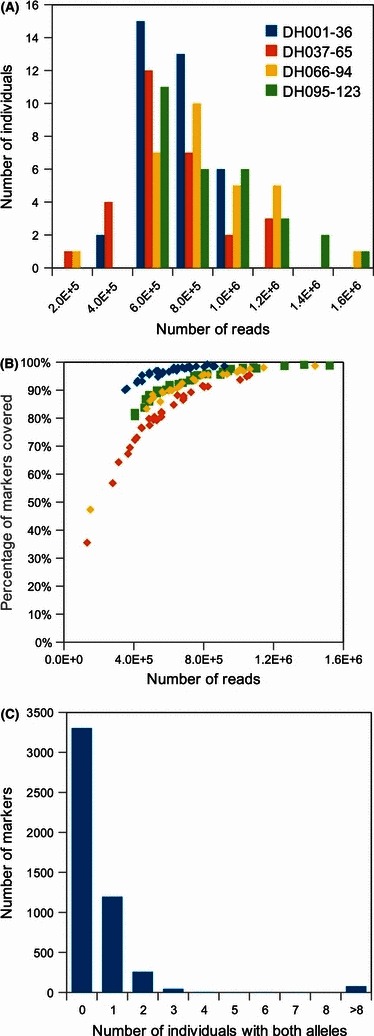
Single nucleotide polymorphisms (SNP) genotyping using next-generation sequencing. (A) Histogram showing the number of filtered reads generated per individual. The four restriction-site associated DNA (RAD) libraries are shown separately in different colours. (B) Scatter plot showing the relationship between the number of reads per individual and the percentage of the 4888 WR × Sw polymorphic markers that have at least one sequence read in that individual. (C) Histogram showing the number of doubled haploid progeny with both alleles present for the polymorphic markers.

For the purpose of genotyping, we sequenced RAD tags from 123 doubled haploid progeny that were generated from a WR × Sw F1 hybrid through androgenesis. We generated four multiplexed RAD libraries with 29–36 barcoded individuals each, produced 18.8–23.5 million filtered reads per library and used the barcodes to identify and separate the reads from each individual (Table 2). Across all four libraries, an average of 676289 reads belonged to each individual. Importantly, the reads were relatively evenly distributed across individuals with 93.4% (115/123) having >400000 reads (Fig. 2A, Table 2).

**Table 2:** Restriction-site associated DNA sequencing results from WR × Sw recombinant doubled haploid progeny

Library name	Number of individuals	Filtered reads	Reads per individual	Marker coverage	CV of marker coverage (%)
DH001-36	36	23454535	614732 ± 150010	6.42 ± 4.07	63.45
DH037-65	29	18859998	586349 ± 221280	5.48 ± 5.43	98.99
DH066-94	29	22701864	749823 ± 254616	7.81 ± 6.31	80.82
DH095-123	29	23190572	769112 ± 292612	8.10 ± 6.49	80.18

Marker coverage, average number of reads per WR × Sw polymorphic locus per individual; CV, coefficient of variation; Sw, Swanson; WR, Whale Rock. ± represents standard deviation.

We next investigated the utility of these data for genotyping by aligning the reads from each individual to the 4888 WR × Sw polymorphic loci. For each individual, we counted the number of reads that matched either allele at each marker and determined the percentage of markers that contained at least one read. Interestingly, the relationship between read number and marker coverage varied between the four multiplexed libraries, with library DH001-36 performing the best and library DH037-65 the worst (Fig. 2B). Library quality was not correlated with the average number of reads per marker but instead was negatively correlated with the variation in reads per marker (high quality correlated with low variation; Table 2). Despite this variation, an average of more than 90% of loci were covered by at least one read per individual, and only low coverage is needed owing to the progeny’s homozygous nature. We conclude that these data are sufficient for thoroughly genotyping most individuals.

The common ancestor of salmonids experienced a whole-genome duplication, and modern species retain most duplicate regions and experience residual tetrasomic inheritance (Allendorf & Thorgaard 1984; Allendorf & Danzmann 1997; Palti *et al.* 2004). Consequently, SNP discovery in salmonids has been plagued with false-positives from paralogous sequences (Smith *et al.* 2005; Sánchez *et al.* 2009; Castaño Sánchez *et al.* 2011; Seeb *et al.* 2011). Therefore, we examined the allele counts at each locus in the doubled haploid progeny to investigate the percentage of SNPs that are from orthologous loci with disomic inheritance. For each marker, the presence of only one parental allele in each individual confirms orthology with disomic inheritance, whereas the presence of both alleles in many individuals could be due to false-positive SNPs from paralogous loci or tetrasomic inheritance. Of the 4888 markers, only 81 (1.7%) had both alleles present in five or more individuals, whereas 3305 (68%) had no individuals with both alleles. The remaining 1502 markers had both alleles in 1–4 individuals, with the vast majority having both alleles in only one or two individuals (Fig. 2C). This pattern is unlikely to be caused by paralogous loci or tetrasomic inheritance and is most likely from residual maternal nuclear DNA, barcode jumping or sequencing error. We conclude that the vast majority of SNPs are from orthologous loci with disomic inheritance and that next-generation sequencing of RAD tags is a powerful approach for SNP discovery in salmonids.

### Genotyping results produce an accurate genetic map

**Fig. 3:**
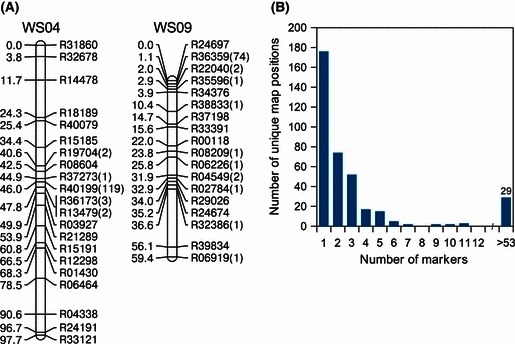
Overview of the WR × Sw genetic map. (A) Two example linkage groups from the WR × Sw (WS) genetic map. Genetic distances are Kosambi centimorgans. Locus names are shown for the subset of marker loci used in final ordering. The number of additional markers at each position is shown in parenthesis. (B) Histogram showing the number of markers per unique map position.

To determine the relative genomic positions of the markers, we converted the allele counts to genotypes (Appendix S2, Supporting information) and constructed a linkage map (see Materials and methods). 4563 markers mapped to 29 linkage groups and with a total map size of 1784.6 cM (Fig. 3A, Fig. S1, Appendix S3, Supporting information). This is the expected number of linkage groups based on the karyotypes of Sw (2*n* = 58) and WR (2*n* = 64) because two acrocentric chromosomes generated through a fission event pair with the homologous fused metacentric chromosome (Ristow *et al.* 1998; Phillips *et al.* 2005). The map consisted of 377 unique positions with an average of 12 markers per position. The average distance between unique positions was 4.73 cM and between markers was 0.39 cM. A genetic map with multiple markers per position was expected from the combination of a large marker number and the analysis of relatively few progeny that were all generated through androgenesis.

We next examined the distribution in the number of markers per unique map position. 348 of the positions had 1–11 markers each, the remaining 29 had 54–310, and each linkage group contained one position with 54 or more markers (Fig. 3A,B). Previous work has shown that recombination in male salmonids takes place almost exclusively in chromosomal regions near the telomeres as opposed to more centrally located regions (Young *et al.* 1998; Nichols *et al.* 2003). We conclude that the 29 positions with 54 or more markers correspond to the large and more centrally located region of each chromosome that lacks recombination in males and that the remaining positions correspond to the regions near telomeres with recombination in males.

To identify the corresponding physical chromosomes, to cross-reference the linkage groups to previous maps and to further validate the overall map, we genotyped the progeny with 62 previously mapped microsatellite markers (Rexroad *et al.* 2008; Palti *et al.* 2011) and a PCR-based Y-specific marker (Brunelli *et al.* 2008). We determined the approximate map position of each marker and found a one-to-one correspondence between linkage groups and *O. mykiss* chromosomes (Appendix S4, Supporting information) (Phillips *et al.* 2006; Rexroad *et al.* 2008; Palti *et al.* 2011). The combination of correct linkage group number, expected recombination patterns and correspondence with chromosomes confirms the accuracy of the genetic map. We conclude that next-generation sequencing of RAD tags facilitates rapid production of accurate genetic maps.

### A conserved haplotype controls rapid development in Cl and Sw

**Fig. 4:**
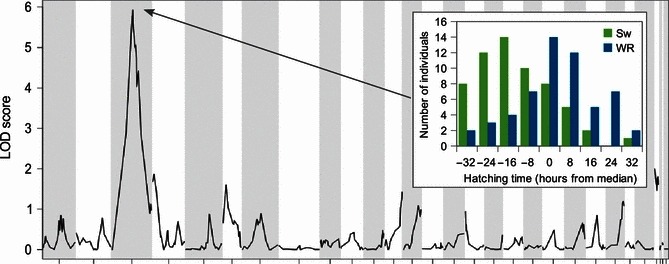
Quantitative trait locus (QTL) analysis of development rate. Development rate logarithm of odds (LOD) profiles for each of the 29 linkage groups. The linkage groups are shown in order, the background is shaded for every other group, and tick marks along the x-axis represent the centre of each group. The inset histogram represents the development rate phenotypic profile for individuals that inherit either the Sw or WR allele at the major QTL peak. [Correction after online publication 28 October 2011: the first word of the caption was corrected to ‘Quantitative’].

To investigate the genetic basis of the differential development rate between Sw and WR, we performed a genome-wide single QTL scan using development rate phenotypes from the doubled haploid progeny (Appendix S2, Supporting information). We identified only one genomic region with a significant influence on development rate. This QTL is located near the centre of linkage group 3 and controls 21.0% of the observed variation (Fig. 4). We next examined the map positions of the microsatellite markers and determined that this region corresponds to the region previously identified in OSU × Cl and OSU × Sw (Table 3) (Robison *et al.* 2001; Nichols *et al.* 2007). These results suggest that the same gene or gene complex is modified either in both OSU and WR causing a decreased development rate or in both Cl and Sw causing an increased development rate.

**Table 3:** Genomic position comparison of the development rate quantitative trait locus (QTL) from WR × Sw and OSU × Cl crosses [Correction after online publication 28 October 2011: QTL was corrected to be ‘quantitative’]

Locus	Palti *et al.* (2011)	OSU × Cl (OC) (Nichols *et al.* 2007)	WR × Sw (WS)
OMM1075	Omy05 16.3 cM	n/a	WS03 2.8 cM
OMYFGT12TUF	Omy05 54.4 cM	OC08 29.0 cM	n/a
OMM1009	Omy05 79.6 cM	OC08 33.7 cM	n/a
OMM5265	Omy05 89.8 cM	n/a	WS03 71.0 cM
Development rate (2-LOD)	n/a	OC08 29.0–37.2 cM	WS03 43.4–80.7 cM

See Nichols *et al.* (2007) for evidence of the overlap between OSU × Cl and OSU × Sw regions.

Cl, clearwater; Sw, Swanson; WR, Whale Rock.

**Fig. 5:**
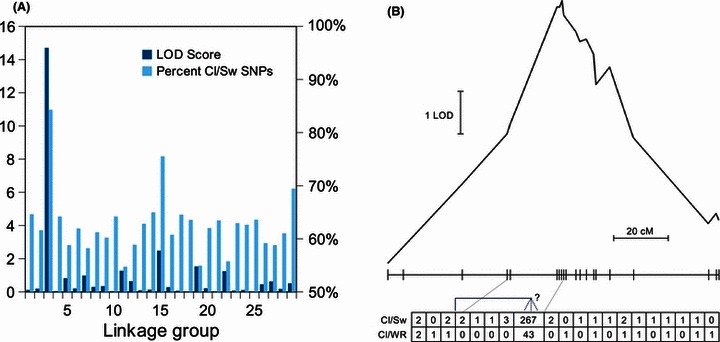
Genomic distribution of Cl/Sw and Cl/WR single nucleotide polymorphisms (SNPs). (A) Histogram showing the percentage and statistical enrichment of SNPs with an allele shared by Cl and Sw (Cl/Sw SNPs) for each linkage group. Logarithm of odds (LOD) scores are from a chi-square test of observed and expected results. (B) Schematic diagram showing the major quantitative trait locus (QTL) peak, linkage group 3 and the number of Cl/Sw and Cl/WR SNPs at each position. Blue bracket represents the enriched region. [Correction after online publication 28 October 2011: QTL was corrected to be ‘quantitative’]

To investigate the potential for a shared allelic basis for rapid development, we examined the genomic distribution of SNPs with an allele shared by Cl and Sw (Cl/SW) or Cl and WR (Cl/WR). Of the 4563 SNPs on our genetic map, 2907 (63.7%) are Cl/Sw and the remaining 1656 (36.3%) are Cl/WR. Strikingly, linkage group 3 was massively enriched (*P*-value = 1.96*10^−15^) for SNPs with an allele shared by Cl and Sw. 84.3% (290/344) of SNPs in this linkage group were Cl/Sw, whereas only 63.7% would be expected by chance (Fig. 5A). Furthermore, this enrichment was not uniform across the chromosome but restricted to the region under the QTL peak near the centre of the linkage group. The limited genetic mapping resolution prevents an exact determination of the conserved area, but the area is clearly large and covers part of the region of one arm with recombination in males and a substantial percentage of the more centrally located region that lacks recombination in males (Fig. 5B). Lastly, this enrichment was much greater than that of any other genomic region or linkage group (Fig. 5A, Appendix S5, Supporting information). Taken together, these results suggest that Cl and Sw share a conserved haplotype that controls rapid development.

## Discussion

### The genetic basis of parallel adaptation in salmonids

The evolution of similar phenotypes in independent but closely related lineages is referred to as parallel phenotypic evolution or parallel adaptation. This process has occurred in a wide range of species and investigating its underlying genetic basis is an exciting area of research (Elmer & Meyer 2011). In principle, three basic genetic patterns could be responsible for the evolution of parallel phenotypes: the same mutation in the same gene; different mutations in the same gene; or different mutations in different genes. Furthermore, any of these genetic patterns could utilize either *de novo* mutation or standing genetic variation (Elmer & Meyer 2011). Identifying the alleles that underlie adaptive variation is a challenging task requiring a combination of approaches, but the handful of current examples has demonstrated the use of all three genetic patterns and both *de novo* mutation and standing variation (Colosimo *et al.* 2005; Bernatchez *et al.* 2010; Chan *et al.* 2010).

Here, we investigated the genetic basis of the parallel phenotypes of rapid development in two *O. mykiss* clonal lines that originated from geographically and genetically distant populations. We used QTL mapping to identify the genomic region responsible for rapid development in the Sw line and found that this region co-localized with the region previously found to be responsible in Cl. We next examined the genomic distribution of SNPs with alleles shared between these two lines and discovered a striking enrichment in the region responsible for rapid development relative to anywhere else in the genome. Even though we have not identified the responsible gene(s) or mutation(s), this combination of results strongly suggests that the parallel rapid development phenotypes evolved through the repeated utilization of a pre-existing haplotype that already contained the responsible mutation(s). This process is analogous to the genetic basis of the repeated phenotypic evolution of armour plate loss in stickleback fish (Colosimo *et al.* 2005; Schluter & Conte 2009).

Although our results provide mechanistic insight into this process, much remains unknown and could be elucidated by future work. For example, characterizing this genomic region in the remaining clonal lines could reveal whether slower developing lines share a haplotype or whether they contain more variation. Furthermore, examining both rapid- and slower-developing natural populations could reveal the geographic distribution and extent of maintenance of the rapid development and/or other haplotypes as well as facilitate the identification of signatures of selection. These and numerous other experiments are amply possible and could produce interesting and insightful results.

Parallel adaptation is common among salmonids with two classic examples from Pacific salmonids being the repeated evolution of beach and stream spawning within sockeye (Wood 1995) and adult run-timing variation within chinook (Waples *et al.* 2004) and steelhead. In both cases, populations with different phenotypes but from the same watershed are genetically more similar than populations with the same phenotypes but from different watersheds (Wood 1995; Waples *et al.* 2004). Based on our results and the fact that anadromy could facilitate the flow of genetic material over great distances (Quinn 2005), we predict that most cases of parallel adaptation in anadromous salmonids (and other highly mobile species for that matter) will be achieved through the repeated utilization of the same adaptive alleles, even though patterns of neutral genetic diversity may reflect the geographic proximity of populations as opposed to their adaptive phenotypes. This hypothesis can be readily tested using genomic technologies similar to those presented here.

### Implications for conservation

The recent, rapid and global decline in biodiversity has left conservation biologists searching for methods to distinguish unambiguous units within species for conservation purposes. Much work has utilized patterns of genetic diversity at a relatively small number of loci to help define these units. However, a limited amount of genetic data often fails to distinguish adaptively distinct populations, and therefore, the additional collection of phenotypic and ecological data is recommended (Utter *et al.* 1993; Fraser & Bernatchez 2001). Unfortunately, collecting phenotypic and ecological data requires extensive resources and can still result in the failure to distinguish adaptively distinct populations because important adaptive phenotypes remain cryptic (such as metabolic or other physiological variation).

Recent and continued advances in genomic technologies are making possible the collection of extensive (or even complete) genetic information from many individuals in many populations of many species. If these data were collected, they will facilitate the identification of signatures of selection and adaptive genetic variation on a comprehensive genome scale (Hohenlohe *et al.* 2010b) and provide great power to distinguish closely related but adaptively distinct populations. Furthermore, identifying the genetic diversity that is important for the adaptation of populations to their local environment could provide a framework for designing supplementation and restoration programmes. For example, one or more populations that contain specific adaptive alleles could be identified and used for supplementing an endangered population or re-introducing individuals into a habitat in which the natural population has gone extinct. Importantly, populations with adaptive compatibilities could be identified on the basis of genetic data, even in the absence of phenotypic information.

The repeated use of adaptive genetic variation across distant geographical areas also underscores the importance of conserving populations throughout a species range for the future adaptability of that species. Particular populations could serve as reservoirs for alleles that become important for the successful adaptation of other populations upon environmental changes. For example, southern populations probably contain alleles that confer adaptation to warmer conditions, and these alleles could be utilized by more northern populations as temperatures increase. Unfortunately, many southern populations are already extinct (National Research Council 1996; Gustafson *et al.* 2007) and others too depressed to provide adequate stray-based gene flow. Thus, the extinction or depression of some populations could hinder the future adaptability of others.

### The molecular basis of development rate variation

As stated earlier, the genes and molecular pathways that control the observed variation in development rate remain unknown. Unfortunately, the region controlling development rate has unusually low recombination rates in both males and females (Danzmann *et al.* 2005), which inhibits fine-scale mapping. Population genomic approaches (such as association mapping or genome scans), which take advantage of numerous generations of recombination in nature, are another possibility for improving the mapping resolution and implicating the genes underlying adaptive phenotypes (Colosimo *et al.* 2005; Stinchcombe & Hoekstra 2008). However, accurate phenotyping of development rate in a large number of populations or individuals is not trivial. Furthermore, the large haplotype that is conserved between Cl and Sw is likely to also be conserved in other populations. Therefore, utilizing population genomic approaches to identify the genes responsible for development rate variation will be difficult. The best possibility to identify the molecular basis of this development rate variation may be through expression QTL (eQTL) analyses, which has already been used to identify eQTL that co-localize with development rate (Xu *et al.* 2011).

Although we do not know its exact size, the genomic region that is conserved between Cl and Sw appears to be quite large. The best explanation for the conservation of this large haplotype is that the genomic region contains a complex of genes with co-adapted alleles that are maintained together through selection and/or epistatic interactions. The following evidence supports this hypothesis: the major development rate QTL may actually contain two closely linked but independent loci (Nichols *et al.* 2007); a QTL for sexual maturation and spawning date, another optimal timing trait, also maps to this genomic region (O’Malley *et al.* 2003; Danzmann *et al.* 2005; Leder *et al.* 2006); and this genomic region has an unusually low recombination rate in both males and females compared with other regions (Danzmann *et al.* 2005), which may be indicative of a co-adapted gene complex. An alternative possibility is that a recent hard selective sweep led to the fixation of this haplotype in some population, and the haplotype was subsequently transferred into or between these populations and has not yet eroded through generations of recombination.

### Implications for salmonid genomics

The current major limitation in the genomic analysis of salmonids is the lack of a reference genome sequence. We are in the process of sequencing the *O. mykiss* genome, and an independent project to sequence the Atlantic salmon genome is also underway (Davidson *et al.* 2010). The genetic map generated here will be an important resource for anchoring and ordering sequence scaffolds to and within chromosomes. Furthermore, we are continuing to improve the resolution and marker number of RAD-based genetic maps for *O. mykiss* by including female recombination and increasing the number of recombinant individuals. These resources will allow fine-scale genetic analysis in salmonids and facilitate the identification of genes and molecular pathways that underlie phenotypic variation in these fascinating and important creatures.

## Data accessibility

DNA sequences of the RAD tag loci, genotypes and phenotypes of the doubled haploid progeny, and genetic map positions of the mapped markers are available online as Supporting information. Raw DNA sequence data are available from NCBI SRA accession SRP008051.

## Supporting information

**Fig. S1** The entire WR × Sw (WS)genetic map.

**Appendix S1** DNA sequences of the 40649 RAD tag loci. Sequences include the 8 bp SbfI site plus 60 bp of flanking sequence. Each locus is either not polymorphic or has a SNP allele shared by Cl and Sw (Cl/SW), Cl and WR (Cl/WR), or Sw and WR (Sw/WR).

**Appendix S2** Genotypes and phenotypes of the doubled haploid progeny.

**Appendix S3** Genetic map positions of the 4563 mapped RAD tag markers.

**Appendix S4** Genetic map positions of the 63 PCR-based markers.

**Appendix S5** Genomic distribution of the Cl/Sw and Cl/WR SNPs.

**Protocol S1** RAD library construction protocol.
