# Supplementary figures and images for: A conserved haplotype controls parallel adaptation in geographically distant salmonid populations
Source: Mol Ecol. 2012 Jan;21(2):237–49. doi: 10.1111/j.1365-294X.2011.05305.x (PMC3664428; doi:10.1111/j.1365-294X.2011.05305.x)

# WS01

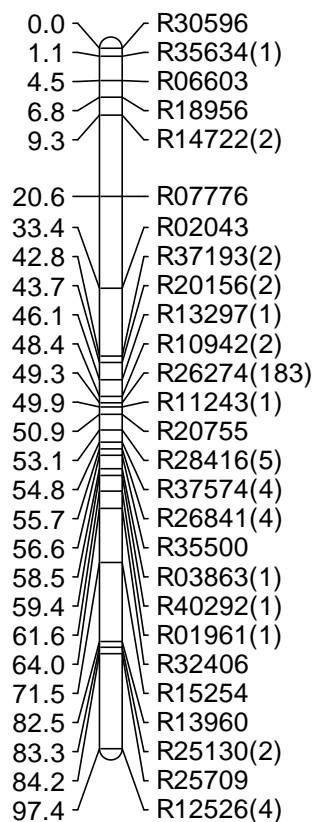

# WS02

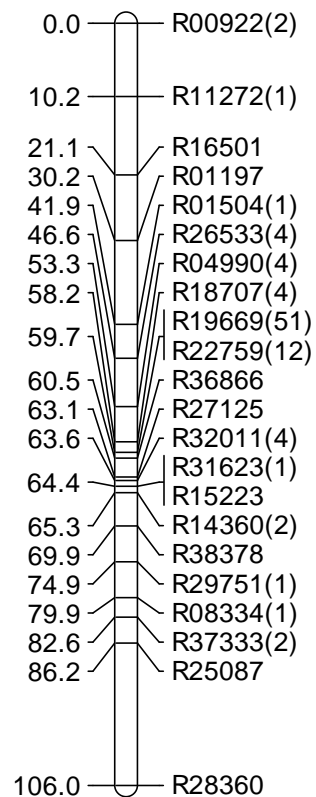

# WS03

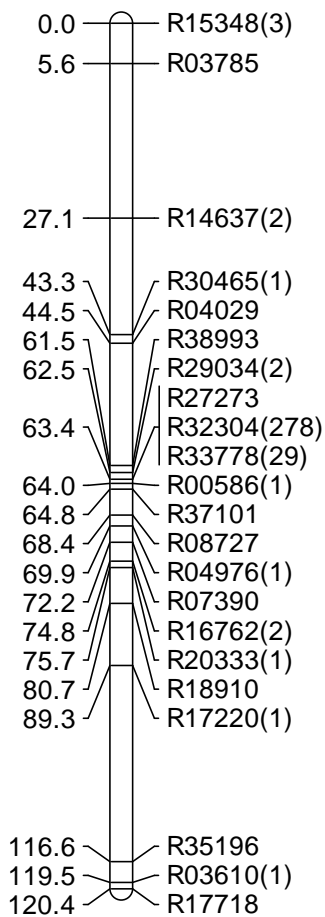

# WS04

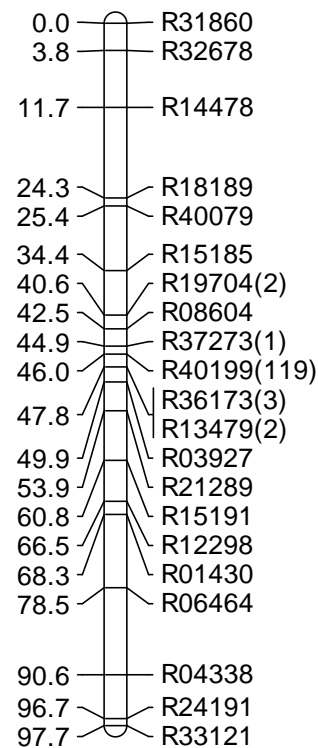

# WS05

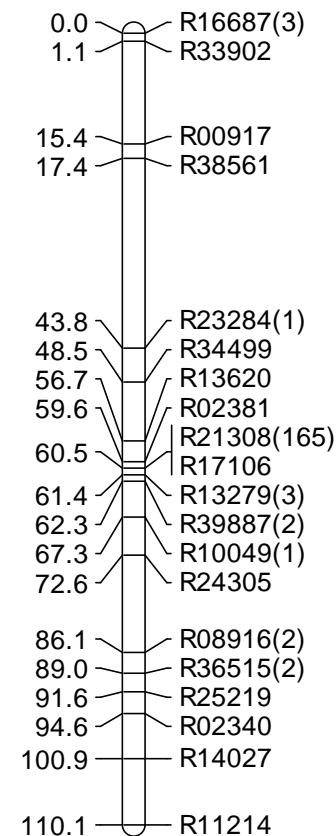

# WS06

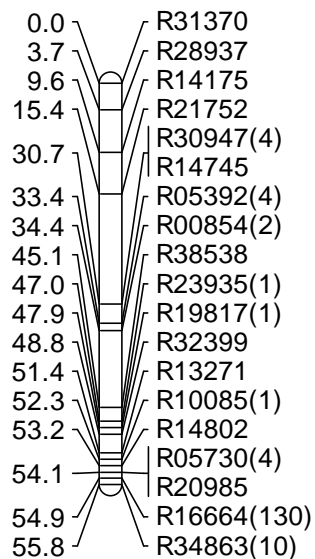

# WS07

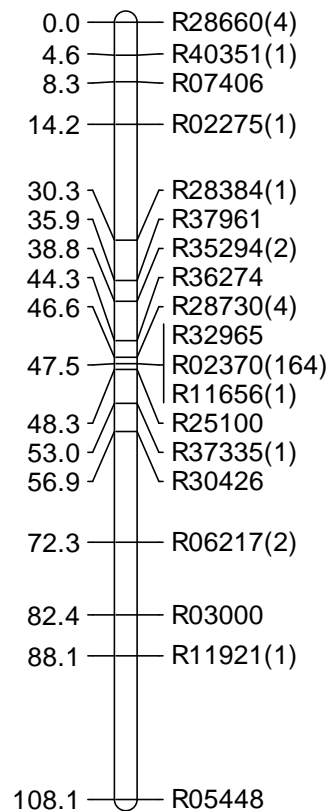

# WS08

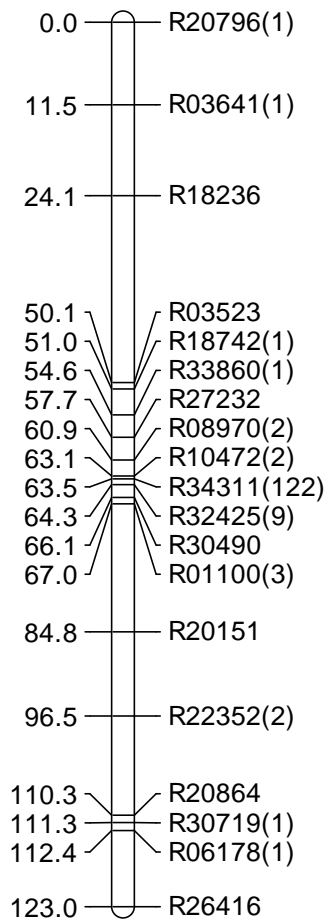

# WS09

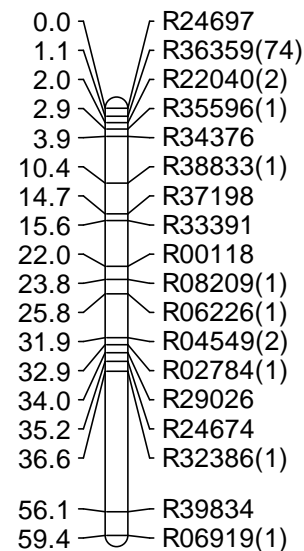

# WS10

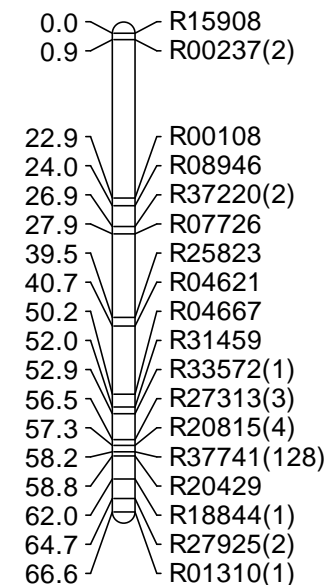

# WS11

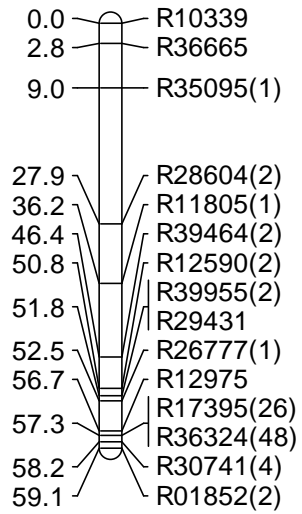

# WS12

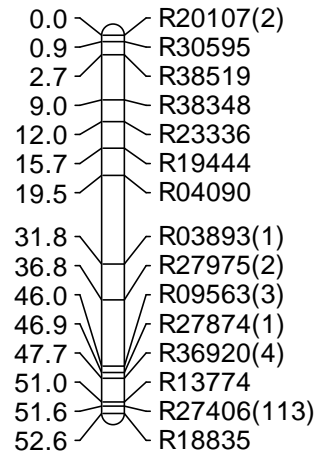

# WS13

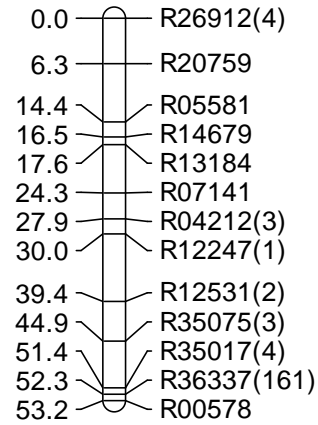

# WS14

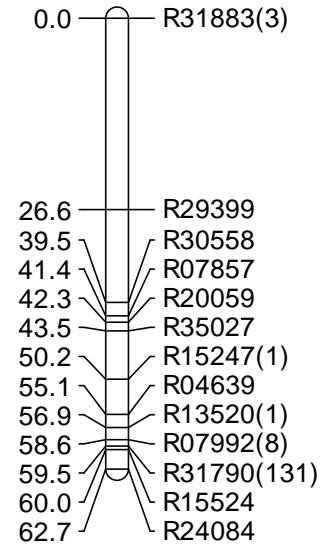

# WS15

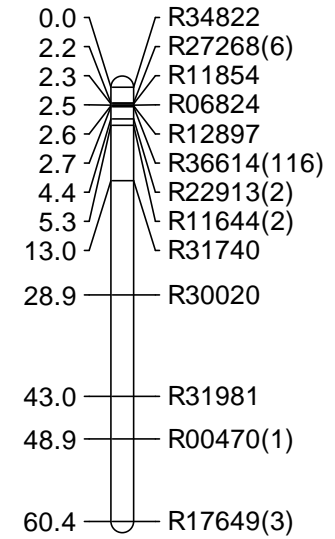

**WS16**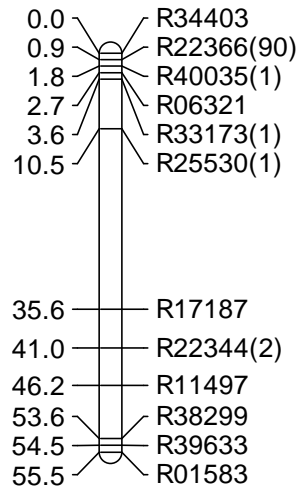**WS17**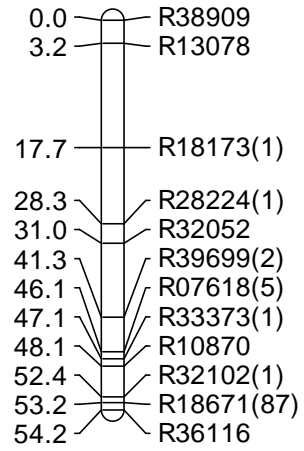**WS18**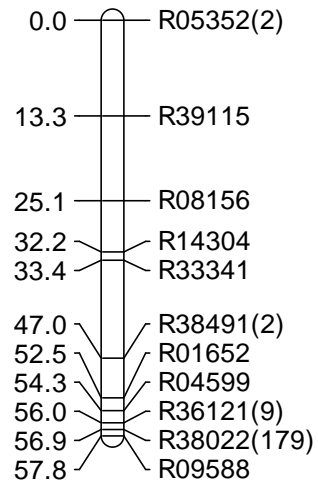**WS19**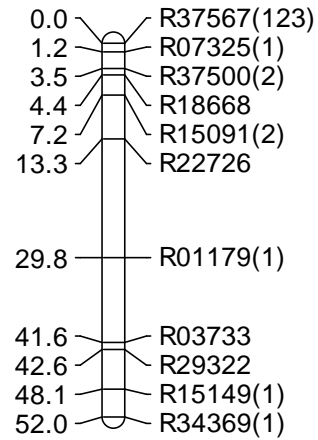**WS20**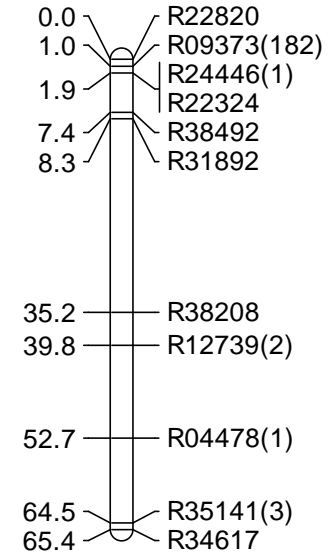

# WS21

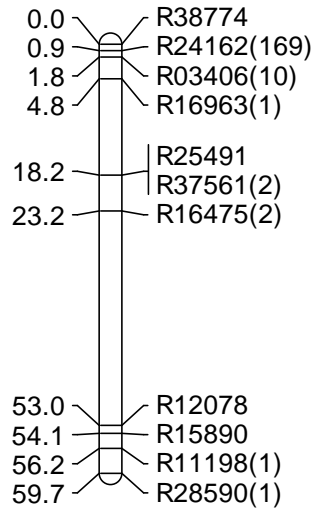

# WS22

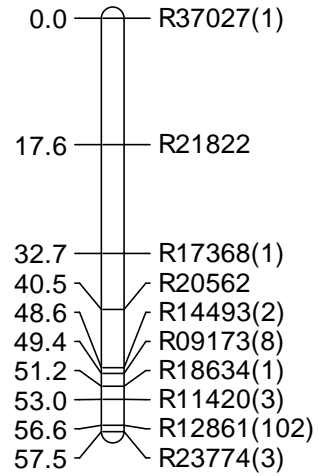

# WS23

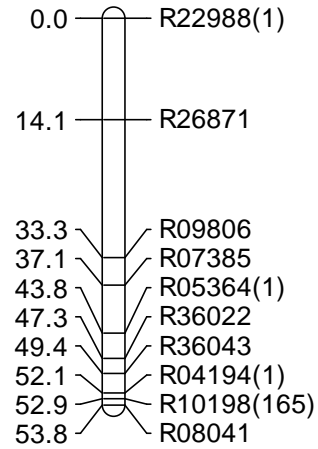

# WS24

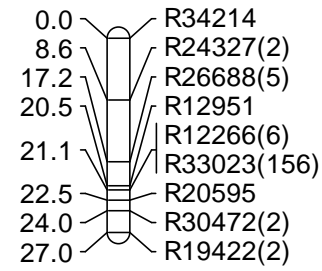

# WS25

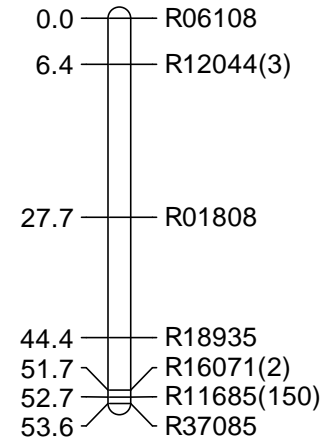

**WS26**

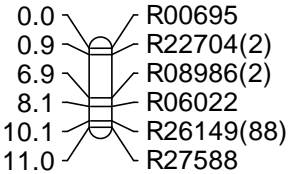

**WS27**

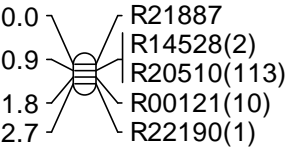

**WS28**

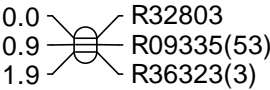

**WS29**

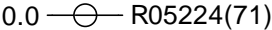

Supplement: Supplementary file 1 [file mec0021-0237-SD1.pdf]
