# Supplementary material for: A conserved haplotype controls parallel adaptation in geographically distant salmonid populations
Source: Mol Ecol. 2012 Jan;21(2):237–49. doi: 10.1111/j.1365-294X.2011.05305.x (PMC3664428; doi:10.1111/j.1365-294X.2011.05305.x)
Supplement: Supplementary file 7 [file mec0021-0237-SD7.pdf]

RAD library construction protocol

1) Start with approximately 500 ng of genomic DNA from each of 96 samples. Bring the volume of each sample to 40 ul with H_20 and transfer the samples into a 96-well plate.
2) Make SbfI digestion mix:

	1X	105X
H_20	4.5 ul	472.5 ul
NEBuffer 4 (10X)	5.0 ul	525 ul
SbfI-HF (NEB R3642L)	0.5 ul	52.5 ul

a) Add 10 ul to each well.
b) Incubate the plate at 37°C for 60 minutes.
c) Incubate the plate at 65°C for 20 minutes to inactivate the enzyme.

3) Add 2 ul of the appropriate barcoded SbfI P1 RAD adapter (50 nM) to each well in the sample plate.
4) Make ligation mix:

	1X	105X
H_20	5.9 ul	619.5 ul
NEBuffer 4 (10X)	1.0 ul	105 ul
rATP (100 mM, Fermentas R0441)	0.6 ul	63 ul
T4 DNA Ligase (NEB M0202M)	0.5 ul	52.5 ul

a) Add 8 ul to each well.
b) Incubate the plate at 20°C for 60 minutes.
c) Incubate the plate at 65°C for 20 minutes to inactivate the enzyme.

5) Multiplex the samples that are to be sequenced together in the same library. For SbfI, we recommend attempting to generate 1.5-2.0 million filtered reads per outbread salmonid sample and much less for doubled haploids. The number of individuals to multiplex will vary greatly depending on the type of Illumina machine, reagent kit, and analysis pipeline. For example, if your machine is getting 25-30 million raw reads per lane, multiplexing 12 samples per lane should give you about 1.5-2.0 million filtered reads per sample. Do this by combining 25 ul from each of 12 plate wells into a 1.5 ml Eppendorf tube. **Every individual that is multiplexed into the same library must have a unique barcode.** Store the plate containing the remaining samples at -20°C.
6) Sonicate the multiplexed sample to produce an average fragment size of 500 bp. We use a Bioruptor on high with cycles of 30 seconds on and 1 minute off for 15 minutes.
7) Purify the sample with the Qiagen MinElute PCR Purification Kit. Elute with 15 ul EB.
8) Gel extract the sample. Run the entire sample on a 1% agarose gel and use a ladder as a reference to extract 400-600 bp fragments. Use Qiagen Buffer QG to dissolve the gel chunk and purify with a Qiagen MinElute column. Elute with 20 ul EB.
9) Blunt end repair the fragments by adding the following reagents to the sample in order:

Blunting buffer (10X)	2.5 ul
dNTP mix (1 mM)	2.5 ul
Blunting Enzyme Mix (NEB E1201L)	1.0 ul

• Incubate at 20°C for 60 minutes.

10) Purify the sample with the Qiagen MinElute PCR Purification Kit. Elute with 43 ul EB.
11) Add A-overhangs to the fragments by adding the following reagents to the sample in order:

NEBuffer 2 (10X)	5.0 ul
dATP (10 mM)	1.0 ul

Klenow Fragment (3' → 5' exo-) (NEB M0212L)	2.0 ul

• Incubate at 37°C for 60 minutes.

12) Purify the sample with the Qiagen MinElute PCR Purification Kit. Elute with 44 ul EB.
13) Ligate the P2 adapter to the fragments by adding the following reagents to the sample in order:

NEBuffer 2 (10X)	5.0 ul
P2 RAD adapter (10 uM)	1.0 ul
rATP (100 mM, Fermentas R0441)	0.5 ul
T4 DNA Ligase (NEB M0202M)	0.5 ul

• Incubate at 20°C for 30 minutes.

14) Purify the sample with the Qiagen MinElute PCR Purification Kit. Elute with 50 ul EB.
15) Make PCR mix:

H_20	38.0 ul
2X Phusion Master Mix (NEB F-531L)	50.0 ul
P1 Adapter Primer (10 uM)	4.0 ul
P2 Adapter Primer (10 uM)	4.0 ul
Purified sample from step 14 (Store remaining at -20°C)	4.0 ul

• Place in thermal cycler and perform PCR. Cycling conditions: 98°C for 30 sec; 14X{98°C 10 sec, 65°C for 30 sec, 72°C for 30 sec}; 72°C for 5 minutes; Hold at 10°C.

16) Purify the sample with the Qiagen MinElute PCR Purification Kit. Elute with 14.5 ul EB.
17) Gel extract the sample to remove the low molecular weight adapter and primer junk. Run the entire sample on a 1% agarose gel and use a ladder as a reference to extract the good PCR product which is around 400-600 bp. Use Qiagen Buffer QG to dissolve the gel chunk and purify with a Qiagen MinElute column. Elute with 14.5 ul EB. Add 1.5 ul of EB containing 1% Tween-20.
18) Quantify the sample with an Invitrogen Qubit. Dilute the sample to 5 or 10 nM with EB containing 0.1% Tween-20 in EB. For example, assuming an average fragment size of 500 bp, the sample should be diluted to 3.25 ng/ul to achieve a final concentration of 10 nM.

SbfI P1 RAD adapter information

Top oligo sequence:
`5' ACACTCTTTCCCTACACGACGCTCTTCCGATCTXXXXXTGC*A 3'`
`*` = Posphorothioate bond
`XXXXX` = 5 bp barcode

Bottom oligo sequence:
`5' /5Phos/YYYYYAGATCGGAAGAGCGTCGTGTAGGGAAAGAGTGT 3'`
`/5Phos/` = 5' Phosphorylation
`YYYYY` = reverse compliment of 5 bp barcode

Annealed adapter sequence:
```
ACACTCTTTCCCTACACGACGCTCTTCCGATCTXXXXXTGC*A
TGTGAGAAAGGGATGTGCTGCGAGAAGGCTAGAYYYYY/5Phos/
```

P1 adapter primer:

5' AATGATACGGCGACCACCGAGATCTACACTCTTTCCCTACACGACGCTCTTCCGATCT 3'

P2 RAD adapter information

Top oligo sequence:
5' /5Phos/GATCGGAAGAGCGGTTCAGCAGGAATGCCGAGACCGATCAGAACA*A 3'

Bottom oligo sequence:
5' CAAGCAGAAGACGGCATACGAGATCGGTCTCGGCATTCCTGCTGAACCGCTCTTCCGATC*T 3'

Annealed adapter sequence:
```
/5Phos/GATCGGAAGAGCGGTTCAGCAGGAATGCCGAGACCGATCAGAACA*A
     T*CTAGCCTTCTCGCCAAGTCGTCCTTACGGCTCTGGCTAGAGCATACGGCAGAAGACGAAC
```

P2 adapter primer:
5' CAAGCAGAAGACGGCATACGA 3'
